# Population structure in *Quercus suber* L. revealed by nuclear microsatellite markers

**DOI:** 10.7717/peerj.13565

**Published:** 2022-06-16

**Authors:** Filipe Sousa, Joana Costa, Carla Ribeiro, Marta Varandas, Francisco Pina-Martins, Fernanda Simões, José Matos, Maria Glushkova, Célia Miguel, Maria Manuela Veloso, Margarida Oliveira, Cândido Pinto Ricardo, Dora Batista, Octávio S. Paulo

**Affiliations:** 1 Faculdade de Ciências, Universidade de Lisboa, cE3c—Centre for Ecology, Evolution and Environmental Changes, Lisboa, Portugal; 2 RAIZ, Herdade de Espirra, Pegões, Portugal; 3 Instituto Nacional de Investigação Agrária e Veterinária, I.P. (INIAV), Unidade de Investigação de Biotecnologia e Recursos Genéticos, Oeiras, Portugal; 4 Polytechnic Institute of Setúbal, ESTBarreiro, Setúbal, Portugal; 5 Forest Research Institute of B.A.S., Department of Forest Genetics, Physiology and Plantations, Sofia, Bulgaria; 6 Faculdade de Ciências, Universidade de Lisboa, Biosystems & Integrative Sciences Institute, Lisboa, Portugal; 7 iBET, Oeiras, Portugal; 8 Universidade Nova de Lisboa (ITQB-NOVA), Instituto de Tecnologia Química e Biológica António Xavier, Oeiras, Portugal; 9 Instituto Superior de Agronomia, Universidade de Lisboa, LEAF—Linking Landscape, Environment, Agriculture and Food (LEAF), Lisboa, Portugal

**Keywords:** Cork oak, Population genetics, Glacial refugia, Conservation, West Mediterranean

## Abstract

*Quercus suber* L. is a sclerophyllous tree species native to the western Mediterranean, a region that is considered highly vulnerable to increased temperatures and severe dry conditions due to environmental changes. Understanding the population structure and demographics of *Q. suber* is essential in order to anticipate whether populations at greater risk and the species as a whole have the genetic background and reproductive dynamics to enable rapid adaptation. The genetic diversity of *Q. suber* has been subject to different studies using both chloroplast and nuclear data, but population structure patterns remain unclear. Here, we perform genetic analyses on *Q. suber* using 13 nuclear microsatellite markers, and analysed 17 distinct locations across the entire range of the species. Structure analyses revealed that *Q. suber* may contain three major genetic clusters that likely result from isolation in refugia combined with posterior admixture and putative introgression from other *Quercus* species. Our results show a more complex structure scenario than previously inferred for *Q. suber* using nuclear markers and suggest that different southern populations contain high levels of genetic variation that may contribute to the resilience of *Q. suber* in a context of environmental change and adaptive pressure.

## Introduction

The geographical distribution, demography and genetic diversity of European tree species have been shaped by environmental oscillations along the Quaternary period, with alternating expansions and contractions for both warm and cold adapted species (Stewart et al., 2010; de Dato et al., 2020). Understanding the mechanisms behind tree species survival through past environmental changes and the current genetic profiles of tree populations is essential in order to predict how species will respond to future climatic oscillations (Petit, Hu & Dick, 2008; Rellstab et al., 2016; Müller & Gailing, 2019). This is especially important as the current pace of environmental changes, together with anthropogenic disturbance and fragmentation of forest ecosystems, may have a severe negative impact on the adaptive capacity of tree species in the near future, thus threatening their long-term survival. A cause of particular concern is the highly biodiverse Mediterranean region, which has been classified as strongly susceptible to climate change due to the expectation of higher temperatures and increased drought frequency (IPCC, 2014). In the Mediterranean region, the adaptive capacity of forest ecosystems is considered more limited than in temperate and boreal regions, in part due to disparities in forest management at local scales (Lindner et al., 2010).

Trees are particularly susceptible to climate oscillations, compared to other plant species, since their long life-span and lower evolutionary rates reduce their capacity to rapidly adapt to environmental changes (Smith & Donoghue, 2008; Lindner et al., 2010; Dauphin et al., 2021). Nevertheless, long-distance gene flow *via* pollen is thought to enhance migration and adaptation to climatic changes in tree species and compensate for their long generation-time, particularly in species with continuous geographical distribution (Kremer, 2010; Kremer et al., 2012). Highly diverse gene pools are also expected to facilitate adaptation *via* natural selection (Kremer, 2010). However, limited seed dispersal and fewer possibilities for establishment in filled landscapes can hinder evolutionary responses to rapid environmental shifts, even in species with extensive genetic variation and population-specific traits that reflect latitudinal and altitudinal adaptation (Savolainen, Pyhäjärvi & Knürr, 2007).

*Quercus suber* (L.) is an evergreen sclerophyllous oak species of major ecological and economic importance in the western Mediterranean region, from the Iberian peninsula to southern Italy, and from Morocco to Tunisia. Where it is dominant, it forms a habitat that harbours important numbers of animal species (Aronson, Pereira & Pausas, 2009). Cork, the unique bark of *Q. suber*, has several industrial applications and its production is a major source of income in the regions where the species is native (Vessella et al., 2017). It is assumed that, as with other European and North American oaks (Cannon et al., 2018), the distribution of *Q. suber* contracted into refugia during the last glacial maximum (Vessella, Simeone & Schirone, 2015; Costa et al., 2011), but the effects of this putative contraction on current population genetic signature are not yet fully understood and were likely partially erased by extensive gene flow (Kremer, 2010). The genetic structure observed in *Q. suber* haplotypes has in fact been hypothesized to be a result of genetic drift driven by Oligocene-Miocene continental margin dynamics, rather than by Quaternary oscillations (Magri et al., 2007).

In the Mediterranean basin, long-term and acute drought stress have been identified as major contemporary drivers of oak decline (Gentilesca et al., 2017). The negative impact of drought stress is particularly concerning in the case of *Quercus suber* because it is considered the most selective of the Mediterranean evergreen oaks regarding temperature, rainfall and soil (Aronson, Pereira & Pausas, 2009; Vessella et al., 2017), and future warmer and drier conditions are expected across its natural range (Vessella et al., 2017). Characterising the genetic diversity and structure of *Q. suber* across its entire range, and identifying drought-tolerant populations as well as genetic profiles and phenotypic traits associated with drought response (Ramírez-Valiente et al., 2010a) will allow for the assessment of the adaptive capacity of different populations. In cases where genetically eroded local populations are at greater risk of non-adaption, assisted seed or seedling transfer may provide additional adaptive buffer capacity (Kremer, 2010). Several recent studies have analysed genetic diversity at population level in *Q. suber*, using nuclear loci (Modesto et al., 2014), genotype-by-sequencing (Pina-Martins et al., 2019; Vanhove et al., 2021), chloroplast microsatellites (Magri et al., 2007) and chloroplast sequence data (López de Heredia et al., 2007a; Costa et al., 2011). Studies using nuclear markers have shown that *Q. suber* is essentially unstructured (Pina-Martins et al., 2019) or weakly structured (Vanhove et al., 2021), whereas chloroplast markers have suggested a division of *Q. suber* into two (Costa et al., 2011) or five lineages (Magri et al., 2007).

Analyses of nuclear microsatellites provide a valuable additional line of evidence to characterize the genetic diversity and structure in *Quercus suber* (López-Aljorna et al., 2007; Soto, Lorenzo & Gil, 2007; Lorenzo et al., 2009; Ramirez-Valiente et al., 2009). Microsatellites, or short sequence repeat (SSR) markers, have been widely employed in population studies because of their high level of intraspecific multistate polymorphism, high mutation rates and co-dominant inheritance (Zhang & Hewitt, 2003; DeFaveri et al., 2013). Microsatellites are often used for studies of genetic diversity and population structure, for example in plants (Zhang et al., 2019; Islam et al., 2020; de Dato et al., 2020), although generating large amounts of single nucleotide polymorphism (SNP) data has become a standard option in many population genetics studies (*e.g.*, Pina-Martins et al., 2019; Feng et al., 2020; Cai et al., 2020; Vanhove et al., 2021). Microsatellites are particularly useful for detecting weak contemporary differences and shallow structuring among populations, and thus for inferring information on gene flow among populations on a fine spatial scale, despite having a higher genotyping error rate and low density across genomes, compared to SNPs (DeFaveri et al., 2013).

Here, we investigate genetic diversity and structure in *Quercus suber* using 13 nuclear SSR loci and extensive sampling within each of 17 populations. We scored diversity metrics at locus and population levels and perform structure analyses using different methods. By comparing our results with earlier population studies in *Q. suber* we interpreted the past demographic history of the species and briefly elaborate on future directions for conservation and management of cork oak given the current knowledge on the distribution of its genetic diversity.

## Materials & Methods

### Sampling and DNA extraction

**Figure 1:**
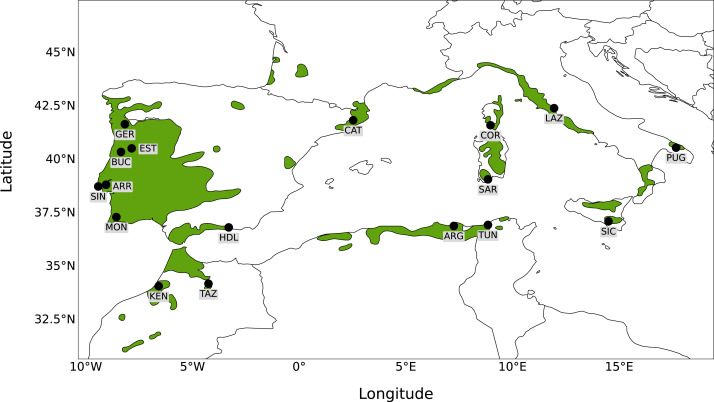
Map of sampling sites. Map showing the distribution of *Quercus suber* around the western Mediterranean and the location of the 17 sampling sites.

Seventeen sampling sites were selected to broadly represent the entire distribution of *Quercus suber* around the Mediterranean. Leaf material from six sampling sites was collected *in situ* from natural stands (Portugal: Gerês, Serra da Estrela, Serra da Arrábida, Serra de Monchique, Serra do Buçaco, Serra de Sintra). For the remaining sites (Spain: Catalonia, Haza del Lino; Italy: Apulia, Lazio, Sardinia, Sicily; France: Corsica; Algeria: Forêt de Guerbès; Tunisia: Mekna ; Morocco: Taza, Kenitra), material was obtained from a cork oak provenance trial (FAIR I CT 95 0202) established in 1998 at Herdade Monte da Fava (Santiago do Cacém, Portugal; 8°7′W, 38°00′N) as part of the European Forest Genetic Resources Programme (EUFORGEN; Varela, 2003). Leaf material was obtained from 22 to 30 individuals from each location, to a total of 488 trees sampled, and kept at −80 °C. Leaves were ground manually using liquid nitrogen and genomic DNA was extracted with the DNeasy Plant Mini Kit (Qiagen, Valencia, CA, USA), according to the manufacturer’s protocol. To determine DNA concentration, quality and integrity, extracted DNA was analysed by gel electrophoresis and with an ND-1000 Nanodrop spectrophotometer. A map showing the 17 sampling sites analysed together with the distribution of *Q. suber* is presented in Fig. 1.

### Microsatellite genotyping

A set of 13 simple sequence repeat (SSR) loci was used for analyses. Of these, ten are anonymous nuclear microsatellites (nuSSR): MSQ13, MSQ4 (Dow, Ashley & Howe, 1995); QpZAG9, QpZAG15, QpZAG36, QpZAG46, QpZAG110 (Steinkellner et al., 1997); QrZAG11, QrZAG7, QrZAG20 (Kampfer et al., 1998) and three are expressed sequence tag microsatellites (EST-SSR): QmOST1, QmD12, QmAJ1 (Ueno & Tsumura, 2008). Polymerase chain reaction (PCR) amplifications were performed in a volume of 15 µL reaction mixture containing 0.5 µL of DNA (50–100 ng), 1x PCR buffer (Promega, Madison, WI, USA), 1U Taq polymerase (Promega, Madison, WI, USA), 1.5(2.0) mM MgCl_2_ , 0.12 mM dNTPs and 0.3(0.4) µM of each primer. Amplifications were performed under the following general conditions: an initial denaturation step at 94 °C for 5 min followed by 30 cycles consisting of denaturation at 94 °C for 30(60) s, annealing at 50(57) °C for 30 s, extension at 72 °C for 30(60)s and a final extension step at 72 °C for 10 min. PCR products were analysed by capillary electrophoresis using the ABI PRISM 310 automated sequencer. Genotypes were scored and visually controlled using GENEMAPPER v3.7 (Applied Biosystems, Inc., Waltham, MA, USA) The software MICRO-CHECKER v2.2.3 (Van Oosterhout et al., 2004) was used to identify and correct possible genotyping errors.

### Genetic diversity analysis

Estimates of inter- and intra-population genetic diversity were calculated. The observed (H_*o*_) and expected (H_*e*_) heterozygosity, the W&C (Weir & Cockerham, 1984) inbreeding coefficient (F_*IS* _), fixation index (F_*ST*_) and the number of observed alleles (Na) per locus were calculated using the GENEPOP package v 1.1.7 (Rousset, 2008) as implemented in R v 3.6.3 (R Core Team, 2013) using the Rstudio environment (R Studio Team, 2016).

F_*IS*_ per locus was estimated considering both the 17 sampling sites separately and the three clusters corresponding to *K* = 3 (see Results) where one cluster contained Lazio, Sardinia, Sicily, a second cluster contained Corsica, Mekna, Guerbès, Apulia and the third cluster contained all other sites. The frequency of null allele (fna) per locus was calculated using PopGenReport (Adamack & Gruber, 2014). Estimates of null allele frequency were obtained with both the method of Chakraborty et al. (1994), and the method of Brookfield (1996). Per-locus diversity statistics were calculated considering all 17 sampling sites as separate populations. Additionally, F_*IS*_ for each population was calculated using GENEPOP and the number of private alleles (Np) and allelic richness (Ar) per population were calculated with PopGenReport v 3.0.4. Pairwise F_*ST*_ across all populations was calculated for the 13 loci and the neutral loci data sets (see “*Tests of non-neutrality”*) with HIERFSTAT (Goudet, 2005), using the Nei 87 (Nei, 1987) genetic distance, and significance was tested with 1,000 bootstraps and a 95% confidence level. A dendrogram of parirwise F_*ST*_ for the neutral loci was constructed from 1,000 bootstrap replicates.

### Tests of Hardy–Weinberg equilibirum and linkage disequilibrium

Deviations from Hardy–Weinberg Equilibrium (HWE) were estimated for the 13 loci and 17 sampling sites using GENEPOP, with the Monte Carlo based exact test for a null hypothesis of random union of gametes, and *p*-values obtained from a global test across samples using Fisher’s method (dememorization = 10,000, batches = 20, iterations = 10,000). Linkage disequilibrium (LD) across all pairs of loci and all 17 sampling sites was tested using GENEPOP with the default Monte Carlo based G-test for the null hypothesis of independence among genotypes at different loci, and the log likelihood ratio as test statistic (dememorization = 10,000, batches = 100, iterations = 5,000).

### Population structure analysis

Structure patterns among sampled populations of *Quercus suber* were inferred using the clustering method implemented in STRUCTURE v 2.3.4 (Pritchard, Stephens & Donnelly, 2000). The program STRUCTURE was wrapped under *Structure_threader* v 1.2.2 (Pina-Martins et al., 2017). An analysis comprising 17 sampling sites and all 13 loci was run with values of K between one and 17, using the admixture model with priors for location information and correlated allele frequencies, a burnin period of 100.000 steps and a post-burning sampling of 500.000 steps, with 20 replicates. The 10 nuSSR loci and the three EST-SSR loci were also analysed separately, using the same parameters as for the analysis of the entire dataset. For each analysis, the best-fitting number of clusters (K) was estimated using the ΔK statistic (Evanno, Regnaut & Goudet, 2005). Finally, an analysis using the 11 “neutral” loci was also performed (see “*Tests of non-neutrality”*), with the same parameters as the first analysis.

Population structure and inference of K was further tested using the program MavericK v 1.0.4 (Verity & Nichols, 2016). The MavericK model differs from the model used in STRUCTURE as it does not include location information nor correlated allele frequencies. Analyses searched for values of K between 1 and 16 using an admixture model with a free alpha parameter of “1”, and comprised five runs of 10.000 iterations with 10% burnin, with thermodynamic integration set to 20 runs of 10.000 iterations and 20% burnin. MavericK was wrapped under *Structure_threader* v 1.2.2 (Pina-Martins et al., 2017). Analyses were run on all 13 loci, on the 10 nusSR and three EST-SSR separately, and on the 11 neutral loci data set.

Principal components analysis (PCA) and correspondence analysis (CA) were performed, respectively, using the methods implemented in the R packages pcaMethods v 1.84 (Stacklies et al., 2007) and ADEGENET v 2.1.3 (Jombart, 2008), on both the 13 loci and 11 neutral loci data sets.

Data files and scripts generated for analyses can be found at https://github.com/CoBiG2/Qsuber_mssats.

### Tests of non-neutrality

Tests of non-neutrality were performed on the 13 SSR loci dataset using the program BAYESCAN v 2.1 (Foll & Gaggiotti, 2008) which assumes that a multinomial Dirichlet distribution can be used to model gene frequencies in neutrally structured populations (Excoffier, Hofer & Foll, 2009; Lotterhos & Whitlock, 2014). To avoid possible violations of the assumptions in the BAYESCAN model causing an excess of false positive outlier loci, and in order to maximise migration within rather than between clusters (Excoffier, Hofer & Foll, 2009), input data was rearranged to comprise two clusters, following the results from STRUCTURE analyses (see Results), rather than 17 groups corresponding to all sampling sites (*e.g.*, Moore et al., 2014). Analyses were run with prior odds for the neutral model of 10, 100 and 1,000, and 20 pilot runs of 5,000 iterations followed by 10,000 iterations and a burnin length of 50,000, with a thinning interval of 10. To confirm the identification of candidate outlier loci, an additional analysis was run considering the results from MavericK analyses that estimated *K* = 3 (see Results), with one cluster comprising the sites Sardinia, Lazio, Sicily, a second cluster comprising the sites Corsica, Apulia, Guerbès, Mekna, and a third comprising the remainder of the sites.

## Results

### Genetic diversity analysis

The 13 SSR loci comprised a total of 145 alleles (Table 1). The number of alleles per locus ranged between 5 (QpD12) and 24 (QpZag110), with an average of 11.2. H_*e*_ ranged between 0.187 (QpZag9) and 0.86 (QpZag110), while H_*o*_ ranged between 0.142 (QpZag9) and 0.777 (QpZag110). The average frequency of null alleles over all 13 loci, inferred with the method of Chakraborty et al. (1994), was 13%, whereas the value inferred with the method of Brookfield (1996), which takes into account the presence of null homozygotes, was 9%. F_*ST*_ per locus ranged between 0.013 (QpZag9) and 0.512 (MSQ13). The mean F_*IS* _ was always positive and ranged between 0.019 (MSQ13) and 0.263 (QmAJ1) when considering the populations separately and between 0.07 (QrZag7) and 0.27 (QmAJ1) when considering *K* = 3 (Table 1).

### Population diversity analysis

Ar ranged between 4.4 and 5.5 among the 17 sites, with the lowest values detected in populations Sicily, Gerês, Sardinia and Corsica, and the largest values found in populations Monchique, Sintra and Guerbès (Table 2). The average observed heterozigosity across 13 loci was lower than expected in all 17 populations. The number of private alleles was zero in five populations (Gerês, Haza del Lino, Kenitra, Apulia, Estrela) and was largest in populations Guerbès and Sintra (six and eight private alleles, respectively) (Table 2). F_*IS* _ varied between 0,046 (Kenitra) and 0,183 (Lazio) (Table 2). Pairwise F_*ST*_ estimates among populations using the Nei 87 method on the neutral data set indicate that sites Lazio, Sicily and Sardinia show low differentiation (F_*ST*_ < 0.05) but differ from the remainder (F_*ST*_ = 0.1–0.24). Corsica also showed differentiation (F_*ST*_ = 0.1–0.21) from all other sites (Table S1).

**Table 1:** Genetic diversity across 13 SSR loci.

Locus	Marker	bp (range)	Na	He	Ho	Fst	fna	Fis
MSQ4	nuSSR	192–218	10	0.677	0.444	0.246	0.207/0.161	0.14/0.19
MSQ13	nuSSR	198–230	12	0.586	0.289	0.512	0.338/0.229	0.02/0.19
QrOst1	EST-SSR	132–152	11	0.622	0.555	0.062	0.057/0.043	0.05/0.09
QpD12	EST-SSR	239–252	5	0.485	0.4	0.108	0.096/0.061	0.08/0.17
QpZag15	nuSSR	101–135	14	0.649	0.460	0.241	0.170/0.129	0.08/0.16
QpZag9	nuSSR	223–249	11	0.187	0.142	0.013	0.135/0.039	0.23/0.24
QpZag46	nuSSR	178–198	9	0.674	0.533	0.082	0.116/0.092	0.14/0.18
QpZag110	nuSSR	208–258	24	0.860	0.777	0.034	0.050/0.046	0.07/0.09
QpZag36	nuSSR	181–225	13	0.847	0.685	0.075	0.105/0.096	0.13/0.17
QrZag20	nuSSR	145–175	7	0.540	0.430	0.046	0.113/0.077	0.17/0.20
QrZag11	nuSSR	255–281	11	0.663	0.594	0.031	0.054/0.043	0.08/0.09
QrZag7	nuSSR	115–133	10	0.789	0.701	0.073	0.058/0.051	0.05/0.07
QmAJ1	EST-SSR	360–385	8	0.645	0.422	0.118	0.208/0.156	0.26/0.27

**Notes.**

The name, type of marker and fragment size range in base-pairs (bp) are indicated for each locus.

Na number of observed alleles He expected heterozygosity Ho observed heterozygosity Fst fixation index fna frequency of null allele estimated with the methods of Chakraborty et al. (1994) (left) and Brookfield (1996) (right) Fis the mean inbreeding coefficient estimated from population estimates with the formula of Weir & Cockerham (1984) for the 17 populations (left) and for K = 3 (right)

**Table 2:** Genetic diversity across 17 sampling sites. For each of the 17 sampling sites, the corresponding country, coordinates, label and number of samples (N) are shown. The mean allelic richness (Ar), number of private alleles (Np), inbreeding coefficient (F_is_), Weir & Cockerham, 1984), expected heterozygosity (H_e_) and observed heterozygosity (H_o_) for each site and 13 SSR loci are presented.

Sampling site	Label	Country	Latitude	Longitude	N	Ar	Np	Fis	He	Ho
Estrela	EST	Portugal	40°32′N	7°51′W	30	4.78	0	0.15	19.40	16.55
Catalonia	CAT	Spain	41°51′N	2°32′E	30	4.51	1	0.05	20.24	19.27
Haza del Lino	HDL	Spain	36°50′N	3°18′W	27	4.53	0	0.11	17.09	15.18
Kenitra	KEN	Morocco	34°05′N	6°35′W	30	4.74	0	0.05	18.85	18.00
Taza	TAZ	Morocco	34°12′N	4°15′W	30	4.76	1	0.1	19.89	17.91
Arrábida	ARR	Portugal	38°50′N	9°03′W	29	4.63	3	0.15	18.37	15.73
Sintra	SIN	Portugal	38°45′N	9°25′W	30	5.35	8	0.09	18.69	17.09
Monchique	MON	Portugal	37°19′N	8°34′W	29	5.3	3	0.14	18.53	15.91
Guerbès	ARG	Algeria	36°54′N	7°15′E	30	5.52	6	0.11	21.11	18.91
Gerês	GER	Portugal	41°40′N	8°10′W	29	4.49	0	0.13	18.17	15.91
Buçaco	BUC	Portugal	40°22′N	8°21′W	30	4.61	1	0.09	18.56	16.91
Mekna	TUN	Tunisia	36°57′N	8°51′E	28	4.77	3	0.1	19.73	17.82
Apulia	PUG	Italy	40°34′N	17°40′E	22	4.76	0	0.15	16.13	13.73
Lazio	LAZ	Italy	42°25′N	11°57′E	27	4.82	1	0.18	16.62	13.64
Sicily	SIC	Italy	37°07′N	14°30′E	29	4.43	2	0.08	19.34	17.73
Sardinia	SAR	Italy	39°05′N	8°51′E	28	4.49	3	0.17	17.55	14.64
Corsica	COR	France	41°37′N	8°58′E	30	4.51	2	0.05	18.40	17.55

### Tests of Hardy–Weinberg equilibirum and linkage disequilibrium

Exact tests of Hardy–Weinberg Equilibrium (HWE) showed an overall departure in all populations (*p* < 0.05) and in all loci except QrZag7 (Table S2). Tests of linkage disequilibrium (LD) for all pairs of loci showed LD (*p* < 0.05) at eight out of 78 pairwise comparisons, when considering 17 sample sites (Table S3).

### Tests of non-neutrality

Tests of non-neutrality using BAYESCAN and considering two clusters (*K* = 2) identified two candidate outlier loci, MSQ13 and QpZag110, for all prior odds (10, 100, 1,000) and with a false discovery rate of 5% (FDR = 0.05). The alpha parameter was positive for MSQ13 (alpha = 1.3091 for prior odds = 10) whereas for QpZag110 alpha was negative (alpha = −1.8945 for prior odds = 10). The additional analysis considering three clusters (*K* = 3) confirmed the identification of the two candidate outlier loci for prior odds of 10 and 100, and FDR =0.05. Plots for the tests of non-neutrality for *K* = 2 and *K* = 3 with prior odds of 10 and FDR = 0.05 are shown in Fig. S1.

### Structure analysis

**Figure 2:**
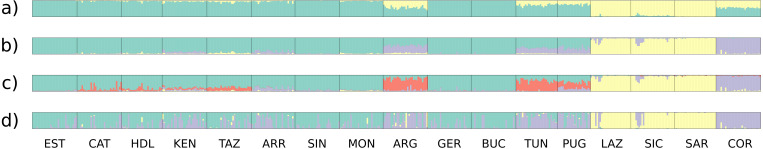
Clustering plots from the analyses of the full data set and the neutral data set. Q-value plots from the STRUCTURE analysis of the 11 neutral loci for *K* = 2 (A), *K* = 3 (B) and *K* = 4 (C); Q-value plots from the MavericK analysis of 11 neutral loci for *K* = 3 (D).

In all analyses using STRUCTURE (13 loci, 10 nuSSR loci, three EST-SSR loci, 11 neutral loci), the ΔK statistic indicated an optimal number of clusters of *K* = 2 (Fig. 2, Fig. S2). In the 11 neutral loci analysis (*K* = 2, ΔK = 123.52), Lazio, Sardinia and Sicily form a separate cluster, whereas populations Apulia, Mekna Guerbès and Corsica show admixture in all individuals. The admixture in Corsica is balanced, with the smallest parental contribution observed in a single individual corresponding to 39.5% (Fig. 2A).

**Figure 3:**
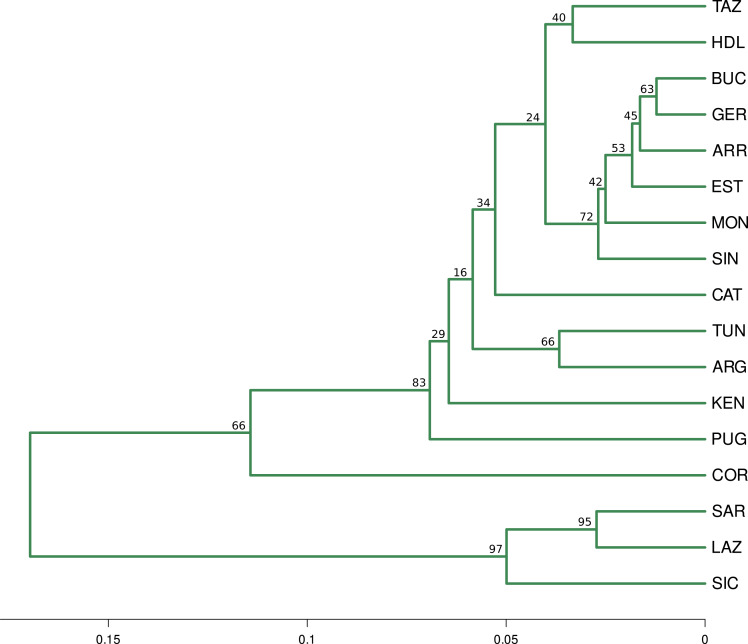
Fst distance dendrogram. Pairwise Fst consensus dendrogram for the 11 neutral loci data set, calculated with the UPGMA method from 1,000 bootstrap replicates.

The optimal number of clusters inferred from the 13 loci, the nuSSR and the EST-SSR datasets, using the thermodynamic integration method implemented on MavericK, was *K* = 2 (Fig. S2). In the 11 neutral loci analysis, the estimated optimal number of clusters was *K* = 3. The Q-matrix plot for *K* = 3 (Fig. 2D) shows that Lazio, Sardinia and Sicily form a distinct cluster without pronounced admixture (except for a few individuals from Lazio and Sicily), and that Corsica forms another cluster with no pronounced admixture. Apulia, Mekna and Guerbès have pronounced admixture and a major genetic contribution from the cluster that includes Corsica. The remainder of the populations form the largest cluster and show admixture that is particularly pronounced in populations Arrábida and Kenitra (Fig. 2D). This same pattern is also seen in the STRUCTURE Q-matrix plot for *K* = 3 (Fig. 2B), whereas the STRUCTURE Q-matrix plot for *K* = 4 assigns Apulia, Mekna and Guerbès to the fourth cluster, while keeping Corsica isolated (Fig. 2C). The pairwise F_*ST*  _ dendrogram (Fig. 3) confirms the clustering of Lazio, Sicily and Sardinia and the separation of Corsica.

**Figure 4:**
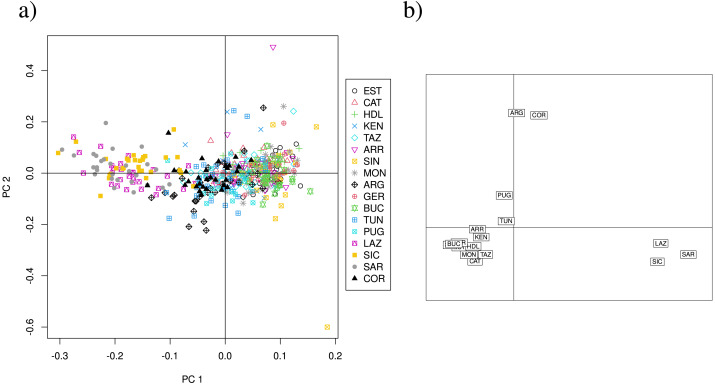
PCA and CA of the 11 neutral SSR loci data set. (A) Principal components analysis (PCA) showing axes 1 and 2; PC1 and PC2 explain 6. 85% of the variance (PC1:4.4%, PC2: 2.45%). (B) Correspondence analysis (CA) showing axes 1 and 2. Population labels as shown in Table 2.

Overall, the PCA using 13 loci does not show a clear separation of clusters, but suggest that Lazio, Sardinia and Sicily form a distinct group, and that Corsica appears separated from the remainder (Fig. S3A), although PC1 and PC2 explain only 6.34% of the variance. The CA of the 13 loci data set separates Lazio, Sardinia and Sicily into one cluster, and isolates Corsica (Fig. S3B). PCA scatterplots of the 11 neutral loci analysis again show a cluster formed by Lazio, Sardinia and Sicily (Fig. 4A), but PC1 and PC2 explain only 6.85% of the variance. The CA scatterplots separate Lazio, Sardinia and Sicily from the remainder of the sites (Fig. 4B), and isolate Corsica and Guerbès.

## Discussion

### Quercus suber nuclear SSR data show heterozygosity deficit and presence of null alleles

The utility of several nuclear SSR markers included in our analyses has been demonstrated in studies of *Quercus suber* diversity (López-Aljorna et al., 2007; Soto, Lorenzo & Gil, 2007; Ramírez-Valiente et al., 2010b), fine-scale structure (Soto, Lorenzo & Gil, 2007; Lorenzo et al., 2009), phenotypic prediction (Ramirez-Valiente et al., 2009) and introgression (Burgarella et al., 2009; López de Heredia, Sanchez & Soto, 2018). Here, we sampled a set of 13 nuclear SSR across 17 locations to infer genetic diversity and structure patterns across the entire natural range of the species. Our analyses of the 13 SSR loci and 17 population data set showed that the observed level of heterozygosity was lower than expected, for all loci, and F_*IS*_ was positive for all loci and samples. The observed presence of null alleles, whether true or apparent, is likely to have strongly contributed to the heterozygosity deficit and positive F_*IS*_ (Waples, 2015). Nevertheless, the frequency of null alleles inferred with the methods of Brookfield and Chakraborty (0.09 and 0.13, respectively) is in line with values commonly reported in the literature and is unlikely to cause a major bias in downstream population structure analyses (Dakin & Avise, 2004). Another possible explanation for low heterozygosity and positive F_*IS*_ is positive assortative mating, of which self-fertilization is an extreme case (Waples, 2015). Selfing is expected to occur to some extent in monoecious species, although *Q. suber* is known to have dichogamous flower development, which promotes outcrossing (Sobral et al., 2020).

Tests of deviation from HWE showed an overall departure that is not specific to particular loci or populations, and linkage disequilibrium (LD) was observed in a small number of different gene pairs. These observed deviations from HWE are not surprising given the presence of null alleles. Furthermore, the species does not conform to the expectations of the Hardy–Weinberg principle, that assumes discrete generations. Like most oaks, *Quercus suber* individuals have great longevity and both natural and managed stands are likely to contain individuals of very different ages. Wind pollination is also likely to promote mating among individuals from distant stands with different ages. In the presence of significant age structure, reproductive cycles do not correspond to random mating among adults of the same generation, but of different generations, each with different allele frequencies, meaning that offspring from different reproductive cycles will differ in their allele frequencies as well. Thus, samples containing mixed-age individuals or their progeny are composed, in practice, of different subpopulations, and both a deficiency of heterozygotes and mixture LD at pairs of loci are expected (Waples, 2015). Error caused by over-sampling of plants with particular phenotypes, for example if early- or late-developing plants are inadvertently preferred, may also cause deviations from HWE, even if the populations themselves are at Hardy–Weinberg equilibrium (Waples, 2015). However, an effect of sampling bias alone is unlikely to explain the HWE departure pattern seen here, which was observed across populations. As the deviations from HWE and sporadic LD in SSR loci are likely a result of reproductive traits of cork oak stands, as well as a consequence of the presence of null alleles, the retention of all 13 loci for downstream analyses was considered justified.

Tests of non-neutrality revealed two outlier loci that are possibly under selection. Locus MSQ13, which had the highest F_*ST*_, had a positive value for the alpha parameter on the BAYESCAN analysis, which could indicate diversifying selection. Locus QpZag110, which had the highest observed heterozygosity and highest number of alleles, had a negative value of alpha, which suggests purifying selection. Outlier detection may result in a high false positive rate when the assumptions of the tests are not met (García-Verdugo et al., 2015). This could be the case of the Dirichlet distribution model in BAYESCAN, which assumes neutrally structured populations (Beaumont, 2005) and is not adequate for species that have undergone range expansion or that display hierarchical population structure (Lotterhos & Whitlock, 2014). Indeed, an exploratory analysis considering all 17 sampling sites as distinct populations resulted in the detection of a very high number of non-neutral loci, which contradicted the expectation that most SSR loci are neutral. Detection of the two outlier loci was attained when the original 17 populations were grouped under two or three clusters. As the detection of the same two outlier loci was consistent across different neutrality prior odds and in the analyses of two and three clusters, the two loci were considered non-neutral, and their exclusion from analyses was deemed justified.

### *Quercus suber* is structured into three population clusters along a longitudinal gradient

The nuclear SSR markers chosen in this study proved to be an effective means for detecting population structure in *Quercus suber*, despite being in much lower number than SNPs . The relative low cost per sample allowed for the inclusion of more samples per location (22 to 30) compared to earlier studies using SNPs, that typically sampled around six individuals per site (Pina-Martins et al., 2019; Vanhove et al., 2021), thus decreasing the probability of biases due to limited sample size.

Analyses of the complete data set, with STRUCTURE and Maverick, and of the neutral data set, with STRUCTURE, show a separation of *Quercus suber* populations into two clusters (*K* = 2) that roughly correspond to an East-West separation. This structural pattern is different to those of Pina-Martins et al. (2019) and Vanhove et al. (2021), based on SNP data. In both those studies, the East-West divide involved a different set of sampling sites and the eastern cluster comprised other sites besides Lazio, Sicily and Sardinia. In contrast, our analysis of the neutral data set using Maverick (*K* = 3) shows a very distinct and homogeneous eastern cluster comprising the central italian population of Lazio and the island populations of Sicily and Sardinia, and a third cluster including Corsica and other sites with pronounced admixture (Apulia, Guerbès and Mekna). The placement of Corsica in a distinct third cluster was also evident in Vanhove et al. (2021).

The hypothesis of *Quercus suber* being structured in three, rather than two clusters, agrees with the pairwise F_*ST*_ calculations, which indicate that populations Lazio, Sicily and Sardinia have significant differentiation from all other populations but not among them, and that population Corsica is distinct from the remainder. Results from PCA and CA analyses also distinguish the group formed by Lazio, Sicily and Sardinia, and separate Corsica from the remainder, often showing a degree of affinity between the latter and populations Apulia, Guerbès and Mekna, which may be a reflection of the admixture pattern for these populations suggested by the Q-plot for *K* = 3. The identification of population Corsica as belonging to a separate cluster, distinct from the eastern and western clusters of *Q. suber* populations, was also hypothesized in the work of Vanhove et al. (2021), with a SNPs dataset. In their work, although the inferred number of K, using bayesian approaches, was also *K* = 2, a shared coancestry matrix assigned individuals from Corsica to a third cluster that is possibly a sub-cluster of the western group. This sub-cluster included populations from southern France (Landes), which are missing from our analyses.

Given the mixed results obtained here with the different methods for estimation of K, it is necessary to consider all evidence in order to ascertain which hypothesis is more likely. Recent studies have shown that the ΔK parameter of Evanno, Regnaut & Goudet (2005) has a tendency to estimate *K* = 2, and strongly recommend against using ΔK alone (Janes et al., 2017). Furthermore, the TI method, which makes use of several closely related MCMC chains to infer K, has been suggested to have a superior performance in this task compared to other methods, and in fact has been shown to outperform ΔK when estimating K from large data sets (Verity & Nichols, 2016). Considering the evidence from pairwise Fst estimates and from PCA and CA analyses, which point to a more complex scenario than a simple separation of *Quercus suber* populations into two groups, and the fact that estimates of *K* = 2 with the ΔK method should be regarded with caution, we posit that a structural pattern of *Q. suber* containing more than two clusters, as estimated by the TI integration method from the neutral loci dataset, is a valid hypothesis.

The possible existence of more than two genetic clusters in *Quercus suber* suggests differentiation caused by isolation in refugia, with posterior expansion and secondary contact between isolated populations. Earlier studies using chloroplast and AFLP markers had already suggested the existence of several glacial refugia and a division of *Q. suber* into three genetic clusters, albeit with different compositions (Lumaret et al., 2005; López de Heredia et al., 2007a; López de Heredia et al., 2007b). The detection of high levels of private alleles may indicate putative refugia, while low genetic diversity indicates newly colonized areas (de Dato et al., 2020). The highest number of private alleles encountered in our data set belong to populations Guerbès and Sintra, while populations Sardinia, Mekna, Monchique and Arrábida also presented an above average number of private alleles. This result is compatible with a scenario of contraction of *Q. suber* into multiple southern refugia during the last glacial maximum, not only in central southern Mediterranean, but also in southwestern Iberian regions (Lumaret et al., 2005; López de Heredia et al., 2007b; Vessella, Simeone & Schirone, 2015). Thus, the hypothesis that genetic differentiation occurred prior to a recent northwards expansion of *Q. suber* seems conceivable.

The observation of an incomplete East-West structure in *Quercus suber* has been interpreted as either the result of a balance between gene flow and local adaptation or the consequence of differential hybridisation, while recent expansion from refugia would only be detected in chloroplast lineages (Pina-Martins et al., 2019). Considering the type of marker used here, essentially neutrally behaving genomic regions which at most segregate passively along with regions under selection, it can be argued that local adaptation is unlikely to account for the observed structure pattern. On the contrary, the more complex scenario involving three genetic clusters was only revealed when loci under selection were excluded from the analysis. This veiling of structure due to selection seems to result from two combined forces. The first is the overall homogenizing effect of balancing selection, where the same alleles are kept in similar frequencies in different populations. The second is directional selection, which promotes identical allele frequencies under similar ecological/climatic circumstances, but differentiates populations in contrasting ecological circumstances, consequently favouring the two cluster scenario. Hybridisation between *Q. suber* and both *Q. ilex* and *Q. cerris* is indeed known to occur in the western and eastern ranges of the species’ distribution, respectively (Lumaret et al., 2005; Magri et al., 2007; Lumaret & Jabbour-Zahab, 2009; López de Heredia, Sanchez & Soto, 2018; López de Heredia et al., 2020). Introgression from related *Quercus* species may have contributed to the survival of *Q. suber* through glaciation periods (López de Heredia, Vázquez & Soto, 2017) and may also have influenced the number of private alleles of certain populations. However, it is unclear how post-glacial and contemporary introgression from these two species alone could explain the persistence of three hypothetical *Q. suber* genetic clusters. Furthermore, the two species known to hybridise with *Q. suber* are also likely to have been affected by recent contraction-expansion cycles (Guzmán et al., 2015; Bagnoli et al., 2016) and it is in fact possible that hybridisation between species occurred already within shared refugia (Bagnoli et al., 2016). Thus, differentiation of *Q. suber* populations in multiple glacial refugia prior to a northwards expansion of the species is perhaps the main cause of the tripartite structure observed with nuclear SSR loci, even if introgression from other species during and after range contraction occurred. While its role in differentiation is not completely clear, local adaptation should nevertheless be expected to occur in *Q. suber* populations, given the latitudinal and altitudinal variation across the distribution of the species. It is also plausible that human activity and migration after the last glacial cycle and to present time may have influenced diversification in *Q. suber* by facilitating dispersal and gene flow. SSR markers do not always reflect genome-wide genetic diversity in natural populations (Väli et al., 2008), but the identification of distinct genetic clusters and genetically diverse populations based on these 13 nuclear SSR markers should nevertheless provide additional evidence on where new allelic variants and possibly adaptive variation may be encountered.

## Conclusions

Our analyses reveal a clear structural pattern in *Quercus suber* populations, suggesting that differentiation along a longitudinal gradient across the distribution of the species existed before its recent northwards recolonisation. Interestingly, high numbers of private alleles confirm the presence of putative glacial refugia for *Q. suber* in southwestern Iberian peninsula. Hypothetical refuge areas are likely to harbour high genetic diversity, and thus have the potential to contribute with genetic variation necessary to ensure that *Q. suber* populations can cope with environmental changes through extensive gene flow and local adaptation. Thus, it is recommendable that populations in these areas are further investigated and that an adequate conservation strategy ensures their long-term survival.Further studies on the demographics and genetic characterisation of *Q. suber* should also be pursued, namely the study of contemporary selection in areas most affected by drought stress, in order to assess the impact of current climatic conditions on *Q. suber* forests.

##  Supplemental Information

10.7717/peerj.13565/supp-1 Supplemental Information 1 Pairwise Fst across 17 sampling sites Fixation index (Fst) pairwise estimates for each sampling site. Estimates were obtained with the WC84 genetic distance (Weir & Cockerham, 1984) for the 11 loci data set (below the diagonal) and for the 13 loci data set (above the diagonal). Non-significant values are marked with (*). Fst values equal or above 0.15 are highlighted in bold.

10.7717/peerj.13565/supp-2 Supplemental Information 2 Hardy–Weinberg exact tests Results of the Hardy–Weinberg exact tests for each population (a) and locus (b) showing the Chi- square value, degrees of freedom (Df) and *p*-values obtained from a global test across samples using Fisher’s method. Values below *p* = 0.05 are considered statistically significant.

10.7717/peerj.13565/supp-3 Supplemental Information 3 Linkage disequilibrium among pairs of loci Results of the linkage disequilibrium among pairs of loci and 17 populations, showing the *p*-values for the G-test with the null hypothesis of independence among genotypes. Values below *p* = 0.05 are considered statistically significant.

10.7717/peerj.13565/supp-4 Supplemental Information 4 Tests on non-neutrality Plots of the BAYESCAN tests of locus non-neutrality for *K* = 2 (a) and *K* = 3 (b) with neutrality prior odds of 10 and a false discovery rate of 5%. Locus 2: MSQ13; locus 8: QpZag110.

10.7717/peerj.13565/supp-5 Supplemental Information 5 Clustering plots from the analyses of the 13 loci, nuSSR and EST-SSR data sets Q-value plots from the STRUCTURE analysis for *K* = 2 with 13 SSR (a), 10 nuSSR (b) and with 3 EST-SSR loci (c); Q-value plots from the MavericK analysis for *K* = 2 with 13 SSR (d), 10 nuSSR (e) and with 3 EST-SSR loci (f).

10.7717/peerj.13565/supp-6 Supplemental Information 6 PCA and CA of the 13 SSR loci data set (A) Principal components analysis (PCA) showing axes 1 and 2; PC1 and PC2 explain 6.34% of the variance. (B) Correspondence analysis (CA) showing axes 1 and 2. Population labels as shown in Table 2.

## Additional Information and Declarations

### DNA Deposition

The following information was supplied regarding the deposition of DNA sequences: SSR genotyping results are available at GitHub: https://github.com/CoBiG2/Qsuber_mssats.

### Data Availability

The following information was supplied regarding data availability: SSR genotyping results are available at GitHub: https://github.com/CoBiG2/Qsuber_mssats.

## References

[ref-1] Adamack AT, Gruber B (2014). PopGenReport: simplifying basic population genetic analyses in R. Methods in Ecology and Evolution.

[ref-2] Aronson J, Pereira JS, Pausas JG (2009). Cork oak woodlands on the edge: ecology, adaptive management, and restoration.

[ref-3] Bagnoli F, Tsuda Y, Fineschi S, Bruschi P, Magri D, Zhelev P, Paule L, Simeone MC, González-Martínez SC, Vendramin GG (2016). Combining molecular and fossil data to infer demographic history of *Quercus cerris*: insights on European eastern glacial refugia. Journal of Biogeography.

[ref-4] Beaumont MA (2005). Adaptation and speciation: what can Fst tell us?. Trends in Ecology and Evolution.

[ref-5] Brookfield JFY (1996). A simple new method for estimating null allele frequency from heterozygote deficiency. Molecular Ecology.

[ref-6] Burgarella C, Lorenzo Z, Jabbour-Zahab R, Lumaret R, Guichoux E, Petit RJ, Soto Á, Gil L (2009). Detection of hybrids in nature: application to oaks (*Quercus suber* and *Q. ilex*). Heredity.

[ref-7] Cai M, Wen Y, Uchiyama K, Onuma Y, Tsumura Y (2020). Population genetic diversity and structure of ancient tree populations of *Cryptomeria japonica* var. sinensis based on RAD-seq data. Forests.

[ref-8] Cannon CH, Brendel O, Deng M, Hipp AL, Kremer A, Kua CS, Plomion C, Romero-Severson J, Sork VL (2018). Gaining a global perspective on Fagaceae genomic diversification and adaptation. New Phytologist.

[ref-9] Chakraborty R, Zhong Y, Jin L, Budowle B (1994). Nondetectability of restriction fragments and independence of DNA fragment sizes within and between loci in RFLP typing of DNA. AmericanJournal of Human Genetics.

[ref-10] Costa J, Miguel C, Almeida H, Oliveira MM, Matos JA, Simões F, Veloso M, Pinto RC, Paulo OS, Batista D (2011). Genetic divergence in Cork Oak based on cpDNA sequence data. BMC Proceedings.

[ref-11] Dakin EE, Avise JC (2004). Microsatellite null alleles in parentage analysis. Heredity.

[ref-12] Dauphin B, Rellstab C, Schmid M, Zoller S, Karger DN, Brodbeck S, Guillaume F, Gugerli F (2021). Genomic vulnerability to rapid climate warming in a tree species with a long generation time. Global Change Biology.

[ref-13] de Dato GD, Teani A, Mattioni C, Aravanopoulos F, Avramidou EV, Stojnic S, Ganopoulos I, Belletti P, Ducci F (2020). Genetic analysis by nuSSR markers of silver birch (*Betula pendula* Roth) populations in their Southern European distribution range. Frontiers in Plant Science.

[ref-14] DeFaveri J, Viitaniemi H, Leder E, Merilä J (2013). Characterizing genic and nongenic molecular markers: comparison of microsatellites and SNPs. Molecular Ecology Resources.

[ref-15] Dow BD, Ashley MV, Howe HF (1995). Characterization of highly variable (GA/CT) n microsatellites in the bur oak, *Quercus macrocarpa*. Theoretical and Applied Genetics.

[ref-16] Evanno G, Regnaut S, Goudet J (2005). Detecting the number of clusters of individuals using the software STRUCTURE: a simulation study. Molecular Ecology.

[ref-17] Excoffier L, Hofer T, Foll M (2009). Detecting loci under selection in a hierarchically structured population. Heredity.

[ref-18] Feng J, Zhao S, Li M, Zhang C, Qu H, Li Q, Li J, Lin Y, Pu Z (2020). Genome-wide genetic diversity detection and population structure analysis in sweetpotato (*Ipomoea batatas*) using RAD-seq. Genomics.

[ref-19] Foll M, Gaggiotti O (2008). A genome-scan method to identify selected loci appropriate for both dominant and codominant markers: a Bayesian perspective. Genetics.

[ref-20] García-Verdugo C, Sajeva M, La Mantia T, Harrouni C, Msanda F, Caujapé-Castells J (2015). Do island plant populations really have lower genetic variation than mainland populations? Effects of selection and distribution range on genetic diversity estimates. Molecular Ecology.

[ref-21] Gentilesca T, Camarero JJ, Colangelo M, Nole A, Ripullone F (2017). Drought-induced oak decline in the western Mediterranean region: an overview on current evidences, mechanisms and management options to improve forest resilience. IForest-Biogeosciences and Forestry.

[ref-22] Goudet J (2005). Hierfstat, a package for R to compute and test hierarchical F-statistics. Molecular Ecology Notes.

[ref-23] Guzmán B, López CMR, Forrest A, Cano E, Vargas P (2015). Protected areas of Spain preserve the neutral genetic diversity of *Quercus ilex* L. irrespective of glacial refugia. Tree Genetics & Genomes.

[ref-24] IPCC (2014). Climate change 2014: impacts, adaptation, and vulnerability. Part A: global and sectoral aspects. Contribution of working group II to the fifth assessment report of the intergovernmental panel on climate change.

[ref-25] Islam MR, Zhang Y, Li ZZ, Liu H, Chen JM, Yang XY (2020). Genetic diversity, population structure, and historical gene flow of *Nelumbo lutea* in USA using microsatellite markers. Aquatic Botany.

[ref-26] Janes JK, Miller JM, Dupuis JR, Malenfant RM, Gorrell JC, Cullingham CI, Andrew RL (2017). The K = 2 conundrum. Molecular Ecology.

[ref-27] Jombart T (2008). adegenet: a R package for the multivariate analysis of genetic markers. Bioinformatics.

[ref-28] Kampfer S, Lexer C, Glossl J, Steinkellner H (1998). Characterization of (GA)n Microsatellite Loci from Quercus robur. Hereditas.

[ref-29] Kremer A (2010). Evolutionary responses of European oaks to climate change. Irish Forestry.

[ref-30] Kremer A, Ronce O, Robledo-Arnuncio JJ, Guillaume F, Bohrer G, Nathan R, Bridle JR, Gomulkiewicz R, Klein EK, Ritland K, Kuparinen A, Gerber S, Schueler S (2012). Long-distance gene flow and adaptation of forest trees to rapid climate change. Ecology Letters.

[ref-31] Lindner M, Maroschek M, Netherer S, Kremer A, Barbati A, Garcia-Gonzalo J, Seidl R, Delzon S, Corona P, Kolström M, Lexer MJ, Marchetti M (2010). Climate change impacts, adaptive capacity, and vulnerability of European forest ecosystems. Forest Ecology and Management.

[ref-32] López-Aljorna A, Bueno MÁ, Aguinagalde I, Martín JP (2007). Fingerprinting and genetic variability in cork oak (*Quercus suber* L.) elite trees using ISSR and SSR markers. Annals of Forest Science.

[ref-33] López de Heredia U, Carrión JS, Jiménez P, Collada C, Gil L (2007b). Molecular and palaeoecological evidence for multiple glacial refugia for evergreen oaks on the Iberian Peninsula. Journal of Biogeography.

[ref-34] López de Heredia U, Jiménez P, Collada C, Simeone MC, Bellarosa R, Schirone B, Cervera MT, Gil L (2007a). Multi-marker phylogeny of three evergreen oaks reveals vicariant patterns in the Western Mediterranean. Taxon.

[ref-35] López de Heredia U, Mora-Márquez F, Goicoechea PG, Guillardín-Calvo L, Simeone MC, Soto Á (2020). ddRAD sequencing-based identification of genomic boundaries and permeability in *Quercus ilex* and *Q. suber* hybrids. Frontiers in Plant Science.

[ref-36] López de Heredia U, Sanchez H, Soto A (2018). Molecular evidence of bidirectional introgression between *Quercus suber* and *Quercus ilex*. IForest-Biogeosciences and Forestry.

[ref-37] López de Heredia U, Vázquez FM, Soto Á (2017). The role of hybridization on the adaptive potential of Mediterranean sclerophyllous oaks: the case of the *Quercus ilex* x *Q. suber* complex. Oaks physiological ecology. Exploring the functional diversity of genus *Quercus* L.

[ref-38] Lorenzo Z, Burgarella C, de Heredia UL, Lumaret R, Petit RJ, Soto A, Gil L (2009). Relevance of genetics for conservation policies: the case of Minorcan cork oaks. Annals of Botany.

[ref-39] Lotterhos KE, Whitlock MC (2014). Evaluation of demographic history and neutral parameterization on the performance of FST outlier tests. Molecular Ecology.

[ref-40] Lumaret R, Jabbour-Zahab R (2009). Ancient and current gene flow between two distantly related Mediterranean oak species, Quercus suber and *Q. ilex*. Annals of Botany.

[ref-41] Lumaret R, Tryphon-Dionnet M, Michaud H, Sanuy A, Ipotesi E, Born C, Mir C (2005). Phylogeographical variation of chloroplast DNA in cork oak (*Quercus suber*). Annals of Botany.

[ref-42] Magri D, Fineschi S, Bellarosa R, Buonamici A, Sebastiani F, Schirone B, Simeone MC, Vendramin GG (2007). The distribution of *Quercus suber* chloroplast haplotypes matches the palaeogeographical history of the western Mediterranean. Molecular Ecology.

[ref-43] Modesto IS, Miguel C, Pina-Martins F, Glushkova M, Veloso M, Paulo OS, Batista D (2014). Identifying signatures of natural selection in cork oak (*Quercus suber* L.) genes through SNP analysis. Tree Genetics & Genomes.

[ref-44] Moore JS, Bourret V, Dionne M, Bradbury I, O’Reilly P, Kent M, Chaput G, Bernatchez L (2014). Conservation genomics of anadromous Atlantic salmon across its North American range: outlier loci identify the same patterns of population structure as neutral loci. Molecular Ecology.

[ref-45] Müller M, Gailing O (2019). Abiotic genetic adaptation in the Fagaceae. Plant Biology.

[ref-46] Nei M (1987). Molecular evolutionary genetics.

[ref-47] Petit RJ, Hu FS, Dick CW (2008). Forests of the past: a window to future changes. Science.

[ref-48] Pina-Martins F, Baptista J, Pappas Jr G, Paulo OS (2019). New insights into adaptation and population structure of cork oak using genotyping by sequencing. Global Change Biology.

[ref-49] Pina-Martins F, Silva DN, Fino J, Paulo OS (2017). Structure_threader: An improved method for automation and parallelization of programs structure, fastStructure and MavericK on multicore CPU systems. Molecular Ecology Resources.

[ref-50] Pritchard JK, Stephens M, Donnelly P (2000). Inference of population structure using multilocus genotype data. Genetics.

[ref-51] R Core Team (2013). http://www.R-project.org/.

[ref-52] R Studio Team (2016).

[ref-53] Ramirez-Valiente JA, Lorenzo Z, Soto A, Valladares F, Gil L, Aranda I (2009). Elucidating the role of genetic drift and natural selection in cork oak differentiation regarding drought tolerance. Molecular Ecology.

[ref-54] Ramírez-Valiente JA, Lorenzo Z, Soto A, Valladares F, Gil L, Aranda I (2010b). Natural selection on cork oak: allele frequency reveals divergent selection in cork oak populations along a temperature cline. Evolutionary Ecology.

[ref-55] Ramírez-Valiente JA, Sánchez-Gómez D, Aranda I, Valladares F (2010a). Phenotypic plasticity and local adaptation in leaf ecophysiological traits of 13 contrasting cork oak populations under different water availabilities. Tree Physiology.

[ref-56] Rellstab C, Zoller S, Walthert L, Lesur I, Pluess AR, Graf R, Bodénès C, Sperisen C, Kremer A, Gugerli F (2016). Signatures of local adaptation in candidate genes of oaks (*Quercus* spp.) with respect to present and future climatic conditions. Molecular Ecology.

[ref-57] Rousset F (2008). genepop’007: a complete re-implementation of the genepop software for Windows and Linux. Molecular Ecology Resources.

[ref-58] Savolainen O, Pyhäjärvi T, Knürr T (2007). Gene flow and local adaptation in trees. Annual Review of Ecology, Evolution, and Systematics.

[ref-59] Smith SA, Donoghue MJ (2008). Rates of molecular evolution are linked to life history in flowering plants. Science.

[ref-60] Sobral R, Silva HG, Laranjeira S, Magalhães J, Andrade L, Alhinho AT, Costa MMR (2020). Unisexual flower initiation in the monoecious *Quercus suber* L.: a molecular approach. Tree Physiology.

[ref-61] Soto A, Lorenzo Z, Gil L (2007). Differences in fine-scale genetic structure and dispersal in *Quercus ilex* L. and *Q. suber* L.: consequences for regeneration of Mediterranean open woods. Heredity.

[ref-62] Stacklies W, Redestig H, Scholz M, Walther D, Selbig J (2007). pcaMethods—a bioconductor package providing PCA methods for incomplete data. Bioinformatics.

[ref-63] Steinkellner H, Fluch S, Turetschek E, Lexer C, Streiff R, Kremer A, Burg K, Glössl J (1997). Identification and characterization of (GA/CT) n-microsatellite loci from *Quercus petraea*. Plant Molecular Biology.

[ref-64] Stewart JR, Lister AM, Barnes I, Dalén L (2010). Refugia revisited: individualistic responses of species in space and time. Proceedings of the Royal Society B: Biological Sciences.

[ref-65] Ueno S, Tsumura Y (2008). Development of ten microsatellite markers for *Quercus mongolica* var. crispula by database mining. Conservation Genetics.

[ref-66] Väli Ü, Einarsson A, Waits L, Ellegren H (2008). To what extent do microsatellite markers reflect genome-wide genetic diversity in natural populations?. Molecular Ecology.

[ref-67] Van Oosterhout C, Hutchinson WF, Wills DP, Shipley P (2004). MICRO-CHECKER: software for identifying and correcting genotyping errors in microsatellite data. Molecular Ecology Notes.

[ref-68] Vanhove M, Pina-Martins F, Coelho AC, Branquinho C, Costa A, Batista D, Príncipe A, Sousa P, Henriques A, Marques I, Belkadi B, Knowles LL, Paulo OS (2021). Using gradient Forest to predict climate response and adaptation in Cork oak. Journal of Evolutionary Biology.

[ref-69] Varela MC (2003). Handbook of the EU Concerted Action on cork oak: FAIR 1 CT 95-0202.

[ref-70] Verity R, Nichols RA (2016). Estimating the number of subpopulations (K) in structured populations. Genetics.

[ref-71] Vessella F, López-Tirado J, Simeone MC, Schirone B, Hidalgo PJ (2017). A tree species range in the face of climate change: cork oak as a study case for the Mediterranean biome. European Journal of Forest Research.

[ref-72] Vessella F, Simeone MC, Schirone B (2015). Quercus suber range dynamics by ecological niche modelling: from the Last Interglacial to present time. Quaternary Science Reviews.

[ref-73] Waples RS (2015). Testing for Hardy–Weinberg proportions: have we lost the plot?. Journal of Heredity.

[ref-74] Weir BS, Cockerham CC (1984). Estimating F-statistics for the analysis of population structure. Evolution.

[ref-75] Zhang DX, Hewitt GM (2003). Nuclear DNA analyses in genetic studies of populations: practice, problems and prospects. Molecular Ecology.

[ref-76] Zhang JJ, Wei X, Chai SF, Wang ZF, Akunne T, Wu SH, Yi JH, Wei JQ, Chen ZY (2019). Genetic diversity and population structure of *Garcinia paucinervis*, an endangered species using microsatellite markers. Conservation Genetics.

