# Supplementary material for: Population structure in Quercus suber L. revealed by nuclear microsatellite markers
Source: PeerJ. 2022 Jun 16;10:e13565. doi: 10.7717/peerj.13565 (PMC9206845; doi:10.7717/peerj.13565)
Supplement: Supplemental Information 1 — Fixation index (Fst) pairwise estimates for each sampling site. Estimates were obtained with the WC84 genetic distance (Weir & Cockerham, 1984) for the 11 loci data set (below the diagonal) and for the 13 loci data set (above the diagonal). Non-significant values are marked with (*). Fst values equal or above 0.15 are highlighted in bold. [file peerj-10-13565-s001.pdf]

	EST	CAT	HDL	KEN	TAZ	ARR	SIN	MON	ARG	GER	BUC	TUN	PUG	LAZ	SIC	SAR	COR
**EST**	**X**	0.066	0.034	0.068	0.047	0.018*	0.031	0.012	0.072	0.008*	0.014*	0.062	0.057	**0.259**	**0.225**	**0.261**	**0.204**
**CAT**	0.069	**X**	0.034	0.071	0.043	0.047	0.054	0.066	0.061	0.057	0.060	0.068	0.093	**0.265**	**0.222**	**0.255**	**0.229**
**HDL**	0.027	0.031	**X**	0.045	0.025	0.032	0.044	0.039	0.051	0.023	0.036	0.066	0.083	**0.262**	**0.226**	**0.258**	**0.216**
**KEN**	0.069	0.076	0.042	**X**	0.064	0.043	0.094	0.080	0.089	0.055	0.077	0.088	0.082	**0.271**	**0.238**	**0.262**	**0.219**
**TAZ**	0.046	0.048	0.029	0.071	**X**	0.044	0.055	0.050	0.032	0.028	0.037	0.050	0.055	**0.221**	**0.194**	**0.220**	**0.219**
**ARR**	0.010*	0.053	0.034	0.048	0.052	**X**	0.023	0.014	0.054	0.005	0.010	0.069	0.063	**0.256**	**0.219**	**0.260**	**0.193**
**SIN**	0.034	0.058	0.047	0.105	0.064	0.026	**X**	0.022*	0.056	0.023*	0.014*	0.066	0.077	**0.270**	**0.236**	**0.273**	**0.225**
**MON**	0.010*	0.075	0.040	0.092	0.057	0.017*	0.026	**X**	0.070	0.011	0.017	0.075	0.066	**0.250**	**0.221**	**0.254**	**0.214**
**ARG**	0.079	0.066	0.056	0.096	0.037	0.061	0.065	0.080	**X**	0.060	0.064	0.037*	0.057	**0.197**	**0.168**	**0.192**	**0.186**
**GER**	-0.002*	0.061	0.024	0.061	0.032	0.005*	0.025*	0.012	0.067	**X**	0.001*	0.067	0.059	**0.264**	**0.231**	**0.270**	**0.206**
**BUC**	0.004*	0.067	0.035	0.085	0.042	0.010*	0.013	0.018*	0.073	-0.001*	**X**	0.081	0.069	**0.274**	**0.237**	**0.278**	**0.213**
**TUN**	0.070	0.062	0.054	0.082	0.040	0.058	0.066	0.076	0.033*	0.059	0.079	**X**	0.048	**0.200**	**0.159**	**0.194**	**0.181**
**PUG**	0.061	0.086	0.068	0.079	0.041	0.050	0.077	0.064	0.049	0.049	0.059	0.048	**X**	**0.197**	**0.174**	**0.205**	**0.158**
**LAZ**	**0.224**	**0.210**	**0.198**	**0.221**	**0.152**	**0.195**	**0.231**	**0.207**	0.135	**0.211**	**0.227**	**0.163**	**0.160**	**X**	0.042	0.020	**0.174**
**SIC**	**0.187**	**0.162**	**0.161**	**0.179**	0.126	**0.151**	**0.194**	**0.171**	0.109	**0.172**	**0.185**	0.121	0.128	0.040	**X**	0.020	**0.146**
**SAR**	**0.231**	**0.202**	**0.201**	**0.210**	**0.158**	**0.204**	**0.240**	**0.213**	0.137	**0.222**	**0.236**	**0.164**	**0.168**	0.017*	0.024	**X**	**0.191**
**COR**	**0.162**	**0.173**	0.149	**0.155**	**0.163**	0.116	**0.183**	**0.164**	0.136	0.139	**0.155**	**0.154**	0.109	**0.187**	**0.164**	**0.213**	**X**

Table S1: Pairwise Fst across 17 sampling sites

Estimates were obtained with the with the Nei 87 genetic distance (Nei 1987) for the 11 loci data set (below the diagonal) and for the 13 loci data set (above the diagonal). Non-significant values are marked with (*). Fst values equal or above 0.15 are highlighted in bold.
