# Supplementary material for: Population structure in Quercus suber L. revealed by nuclear microsatellite markers
Source: PeerJ. 2022 Jun 16;10:e13565. doi: 10.7717/peerj.13565 (PMC9206845; doi:10.7717/peerj.13565)
Supplement: Supplemental Information 2 — Results of the Hardy–Weinberg exact tests for each population (a) and locus (b) showing the Chi- square value, degrees of freedom (Df) and p-values obtained from a global test across samples using Fisher’s method. Values below p = 0.05 are considered statistically significant. [file peerj-10-13565-s002.pdf]

a)

population	Chi2	Df	prob
EST	> 84.1710	26	4.70e-08
CAT	48.7060	24	0.002062
HDL	45.2189	24	0.005488
KEN	77.6549	26	4.80e-07
TAZ	59.7231	26	0.000183
ARR	67.7829	26	1.39e-05
SIN	80.9316	26	1.51e-07
MON	> 62.8958	26	< 6.76e-05
ARG	50.4976	26	0.002741
GER	64.0217	26	4.72e-05
BUC	45.6087	26	0.010085
TUN	60.0086	26	0.000167
PUG	> 103.3482	26	< 3.54e-11
LAZ	> 65.2191	24	< 1.13e-05
SIC	> 76.7472	26	< 6.59e-07
SAR	> 69.6300	26	< 7.51e-06
COR	69.5674	24	2.54e-06

b)

locus	Chi2	Df	prob
MSQ4	> 151.6738	34	< 8.37e-17
MSQ13	52.5398	32	0.012499
QrOst1	68.3738	34	0.000427
QpD12	61.2227	34	0.002850
QpZag15	> 120.8699	34	< 1.16e-11
QpZag9	78.1803	28	1.24e-06
QpZag46	102.5394	34	8.48e-09
QpZag110	78.9470	34	1.96e-05
QpZag36	> 153.6483	34	< 3.83e-17
QrZag20	73.8032	34	9.10e-05
QrZag11	62.1142	34	0.002271
QrZag7	30.1289	34	0.657922
QmAJ1	> 149.9996	34	< 1.62e-16

Table S2: Hardy-Weinberg exact tests

Results of the Hardy-Weinberg exact tests for each population (a) and locus (b) showing the Chi-square value, degrees of freedom (Df) and p-values obtained from a global test across samples using Fisher’s method. Values below $p=0.05$ are considered statistically significant.
