# Supplementary material for: Population structure in Quercus suber L. revealed by nuclear microsatellite markers
Source: PeerJ. 2022 Jun 16;10:e13565. doi: 10.7717/peerj.13565 (PMC9206845; doi:10.7717/peerj.13565)
Supplement: Supplemental Information 3 — Results of the linkage disequilibrium among pairs of loci and 17 populations, showing the p-values for the G-test with the null hypothesis of independence among genotypes. Values below p = 0.05 are considered statistically significant. [file peerj-10-13565-s003.pdf]

	MSQ4	MSQ13	QrOst1	QpD12	QpZag15	QpZag9	QpZag46	QpZag110	QpZag36	QrZag20	QrZag11	QrZag7	QmAJ1
**MSQ4**	**X**												
**MSQ13**	0.2	**X**											
**QrOst1**	0.02	0.93	**X**										
**QpD12**	0.16	0.64	0.07	**X**									
**QpZag15**	0.03	0.87	0.35	0.07	**X**								
**QpZag9**	0.82	0.28	0.64	0.16	0.41	**X**							
**QpZag46**	0.58	0.28	0.75	0.91	0.65	0.99	**X**						
**QpZag110**	0.41	0.89	0.98	0.66	0.48	0.29	0.89	**X**					
**QpZag36**	0.71	0.93	0.83	0.53	0.61	0.55	<1.43e-09	0.76	**X**				
**QrZag20**	0.46	0.18	0.45	0.08	0.39	0.7	0.04	0.74	0.58	**X**			
**QrZag11**	0.3	0.19	0.2	0.49	0.01	0.01	0.05	0.61	0.46	0.24	**X**		
**QrZag7**	0.39	0.05	0	0.33	<0.002654	0.65	0.91	0.87	0.57	0.37	0.95	**X**	
**QmAJ1**	0.42	0.81	0.03	0.19	0.35	0.54	0.3	0.73	0.74	0.34	0.64	0,09	**X**

Table S3: Linkage disequilibrium among pairs of loci

Results of the linkage disequilibrium among pairs of loci and 17 populations, showing the p-values for the G-test with the null hypothesis of independence among genotypes. Values below p=0.05 are considered statistically significant.
