# Supplementary material for: Population structure in Quercus suber L. revealed by nuclear microsatellite markers
Source: PeerJ. 2022 Jun 16;10:e13565. doi: 10.7717/peerj.13565 (PMC9206845; doi:10.7717/peerj.13565)
Supplement: Supplemental Information 4 — Plots of the BAYESCAN tests of locus non-neutrality for K = 2 (a) and K = 3 (b) with neutrality prior odds of 10 and a false discovery rate of 5%. Locus 2: MSQ13; locus 8: QpZag110. [file peerj-10-13565-s004.pdf]

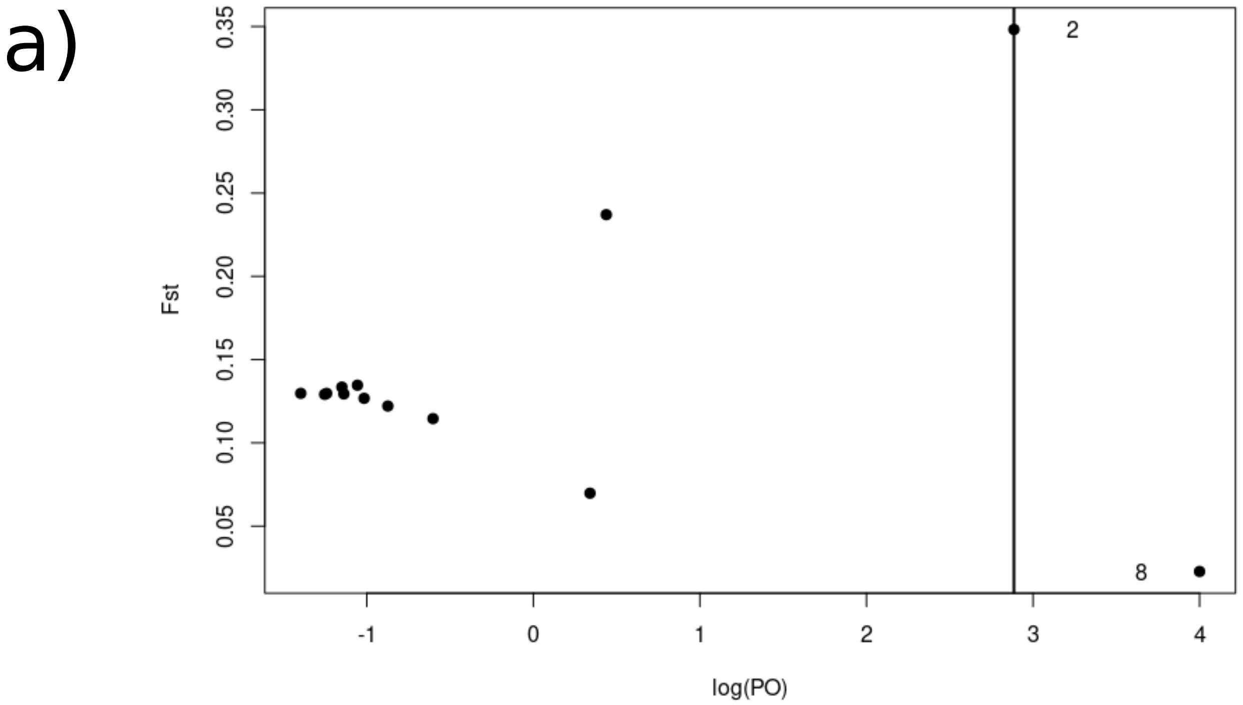

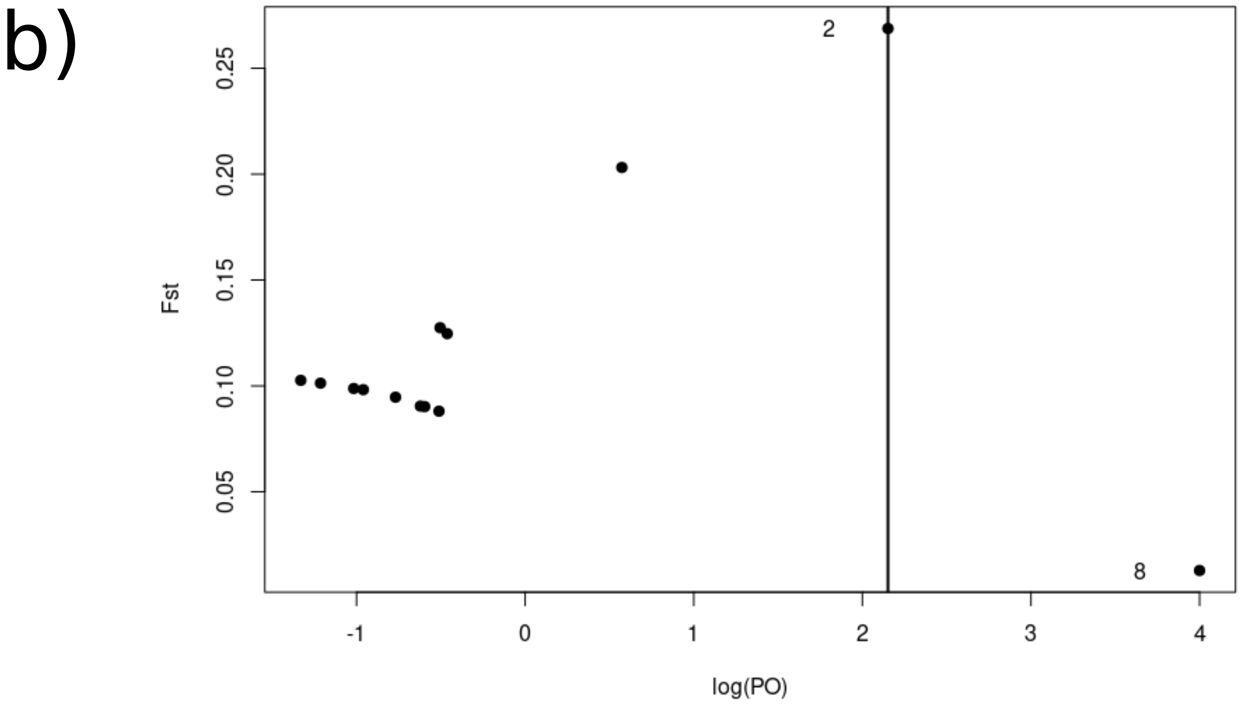

Figure S1: Tests on non-neutrality

Plots of the BAYESCAN tests of locus non-neutrality for K=2 (a) and K=3 (b) with neutrality prior odds of 10 and a false discovery rate of 5%.

The vertical lines indicate the posterior odds (PO) threshold.

Locus 2: MSQ13; locus 8: QpZag110.
