# Supplementary material for: Population structure in Quercus suber L. revealed by nuclear microsatellite markers
Source: PeerJ. 2022 Jun 16;10:e13565. doi: 10.7717/peerj.13565 (PMC9206845; doi:10.7717/peerj.13565)
Supplement: Supplemental Information 5 — Q-value plots from the STRUCTURE analysis for K = 2 with 13 SSR (a), 10 nuSSR (b) and with 3 EST-SSR loci (c); Q-value plots from the MavericK analysis for K = 2 with 13 SSR (d), 10 nuSSR (e) and with 3 EST-SSR loci (f). [file peerj-10-13565-s005.pdf]

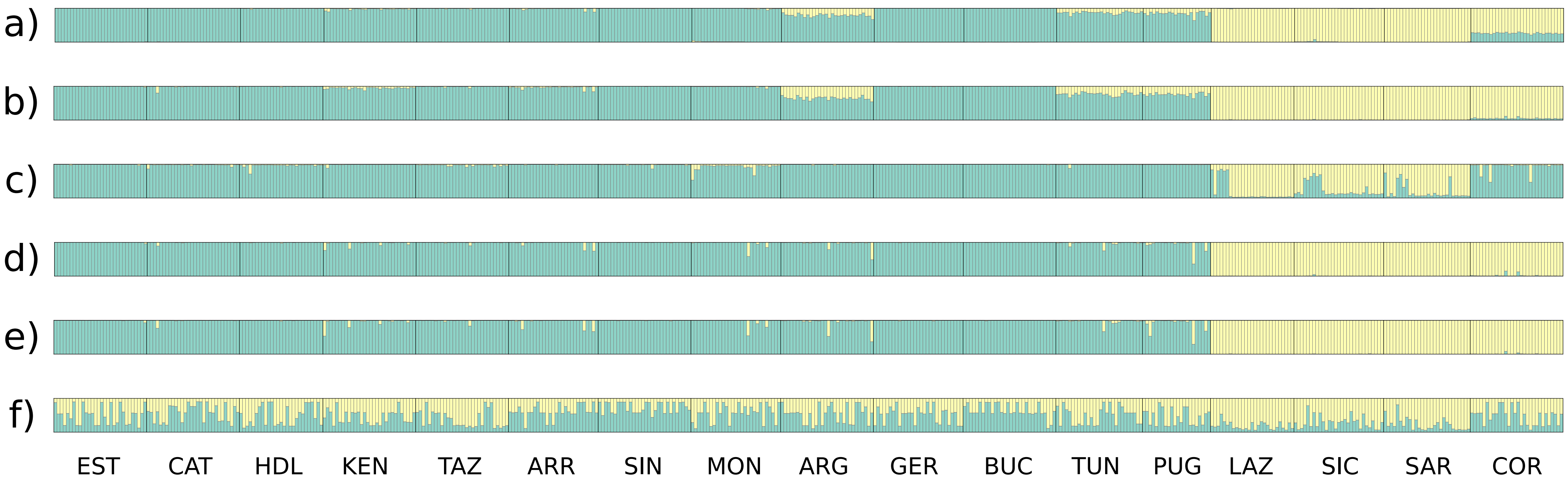

Figure S2: Clustering plots from the analyses of the 13 loci, nuSSR and EST-SSR data sets

Q-value plots from the STRUCTURE analysis for K=2 with 13 SSR (a), 10 nuSSR (b) and with 3 EST-SSR loci (c); Q-value plots from the MavericK analysis for K=2 with 13 SSR (d), 10 nuSSR (e) and with 3 EST-SSR loci (f).
