# Supplementary material for: Population structure in Quercus suber L. revealed by nuclear microsatellite markers
Source: PeerJ. 2022 Jun 16;10:e13565. doi: 10.7717/peerj.13565 (PMC9206845; doi:10.7717/peerj.13565)
Supplement: Supplemental Information 6 — (A) Principal components analysis (PCA) showing axes 1 and 2; PC1 and PC2 explain 6.34% of the variance. (B) Correspondence analysis (CA) showing axes 1 and 2. Population labels as shown in Table 2. [file peerj-10-13565-s006.pdf]

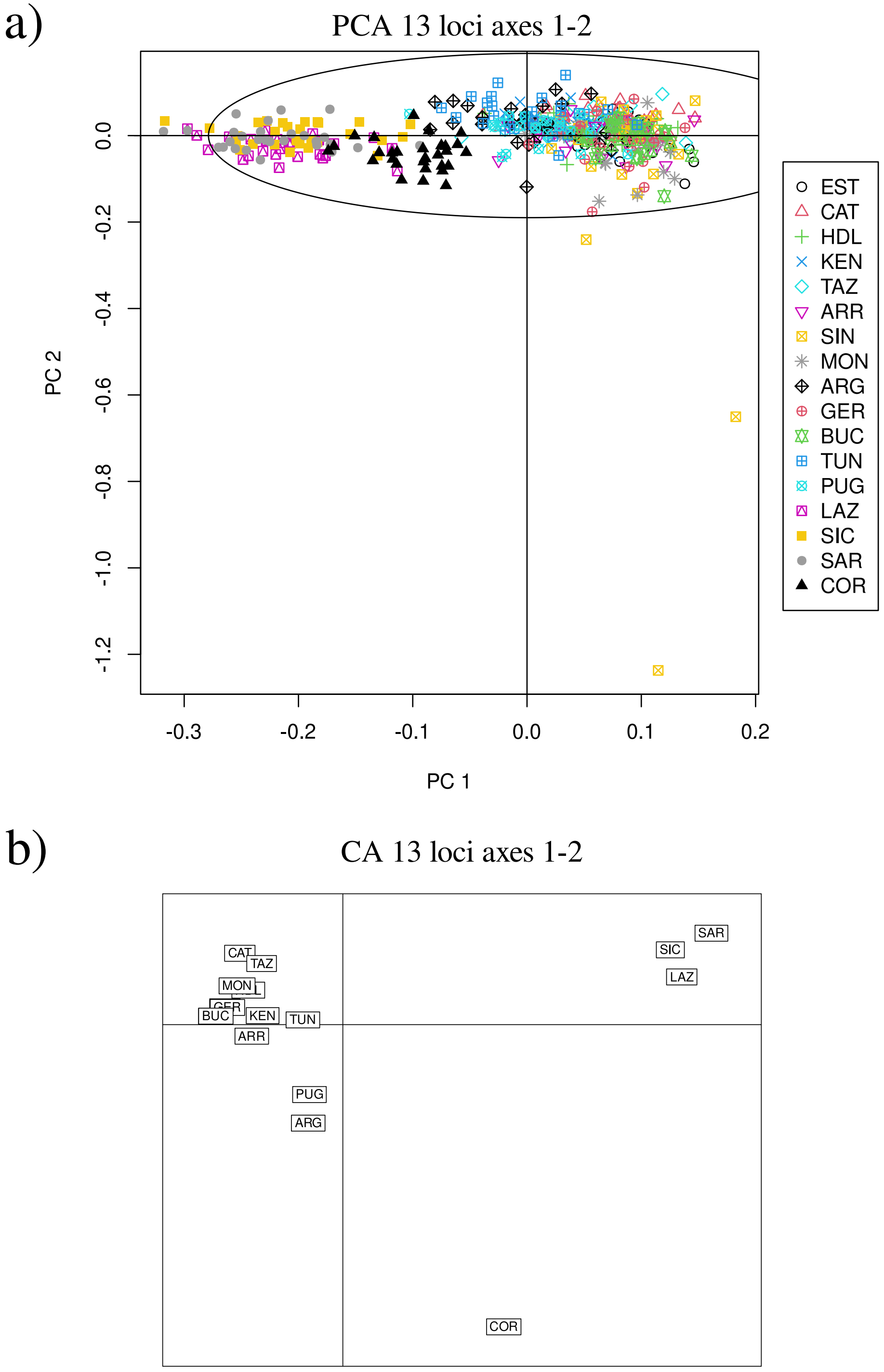

Figure S3: PCA and CA of the 13 SSR loci data set.
a) Principal components analysis (PCA) showing axes 1 and 2; PC1 and PC2 explain 6.34% of the variance (PC1:4.3%, PC2: 2.0%).
b) Correspondence analysis (CA) showing axes 1 and 2.
Population labels as shown in Table 2.
